# Effects of rational emotive behavior therapy on alexithymia, anxiety, depression and sleep quality of older people in nursing homes: a quasi-experimental study

**DOI:** 10.1186/s12912-023-01449-9

**Published:** 2023-08-24

**Authors:** Ning Qin, Jie Li, Xiaoqi Wu, Chun Zhang, Yating Luo, Xiaoqian Dong, Huan Cao, Sha Wang, Min Liu, Jianfei Xie, Andy SK Cheng

**Affiliations:** 1 Nursing Department, The Third Xiangya Hospital, Central South University, Changsha, Hunan 410013 China; 2 Xiang Ya Nursing School, Central South University, Changsha, Hunan 410013 China; 3 Department of Rehabilitation Sciences, The Hong Kong Polytechnic University, Hong Kong, 999077 China

**Keywords:** Rational emotive behavior therapy, Nursing home, Older people, Alexithymia, Anxiety, Depression, Sleep quality

## Abstract

**Background:**

Alexithymia, a subclinical cognitive-affective impairment, is prevalent in older people and increases the risk of mental disorders. There is a vast alexithymia treatment gap, with majority of older people in nursing homes lacking access to adequate mental health care. The study aimed to evaluate the effects of rational emotive behavior therapy (REBT) on alexithymia, anxiety, depression and sleep quality of older people in nursing homes.

**Methods:**

This quasi-experimental study was conducted with two groups (the control group and intervention) from March to November 2021. This study enrolled 86 participants, two of whom were lost to follow-up; 42 received usual care (control group) and 42 received REBT based on usual care (intervention group) in nursing homes. The older people in both groups were evaluated at baseline (T0), within one-week post-intervention (T1), and at 3-month follow-up (T3). Generalized estimating equations were used by SPSS version 26 to assess the differential change in the outcomes between the two groups.

**Results:**

The intervention group shows significantly greater improvement in alexithymia than the control group at both T1 (*β* = -8.167, 95%*CI*= -10.965, -5.368, *P* < 0.001) and T2 (*β*=-4.119, 95%*CI*= -7.171, -1.067, *P* = 0.008). The two groups showed significant differences at both T1 and T2 in both difficulty identifying feelings and difficulty describing feelings. Compared to the control group, the intervention group shows a significant improvement in sleep quality at T2 (*β* = -2.048, 95%*CI*=-4.004, -0.091, *P* = 0.040). The two groups showed significant differences at both T1 and T2 in both sleep disturbance and daytime dysfunction. For depression and anxiety, no significant differences were found between the intervention and control groups.

**Conclusions:**

REBT showed to be an effective method for improving alexithymia and sleep quality of older people in nursing homes. However, it failed to significantly alleviate anxiety and depression at least in a short-term trial. Refining this intervention may have a broader, more substantial impact on future research.

**Supplementary Information:**

The online version contains supplementary material available at 10.1186/s12912-023-01449-9.

## Background

Alexithymia is widespread and sometimes neglected [1]. It refers to a subclinical cognitive-affective impairment that is mainly related with deficiencies to identify, describe and express emotions, manifested most commonly by difficulty identifying feelings, difficulty describing feelings, and extroverted thinking [2]. Several systematic reviews [3, 4] have reported the prevalence of alexithymia to be approximately 10–23% in the general population and 18–50% in psychiatric population. Several lines of evidence have shown that the alexithymia is positively correlated with increasing age, with fewer younger (4.7–27.3%) than older people (13.9–29.3%) [5–8]. The prevalence of alexithymia appears to be higher in China than in other countries [9]. According to a systematic review included 3392 older people Chinese in 2021, the overall prevalence of alexithymia in is 36% (95%*CI*: 30–42%) and the prevalence rate is higher in nursing homes (28–45%) than in the communities (20–38%) [10]. Thus, the prevalence of alexithymia among Chinese older people is particularly worrying. The presence of alexithymia has been linked with an imbalance in an individual’s physical health, mental health and social adaptation. Individuals with alexithymia focus on external changes while ignoring their internal emotions and feelings. They are more prone to anxiety, depression, poor sleep quality, fatigue, non-suicidal self-injury and even suicidal ideation [11–13]. Previous studies have reported that individuals with alexithymia had significant higher anxiety (*β* = 0.54, 95% *CI*: 0.41–0.69, *P* < 0.001), depression (*β* = 0.48, 95% *CI*: 0.34–0.63, *P* < 0.001) [11], and poor sleep quality (*P* < 0.001, *β* = 0.55, 95%*CI*: 0.30–0.79) [12]. Meanwhile, alexithymia can increase the risk of late-life dementia in older people [14] and result in increased complications and a poor quality of life [3]. Therefore, effective and prompt intervention is essential for improving alexithymia among older people in nursing homes.

There is yet no alexithymia-specific guideline, but a number of clinical trials have focused on alleviating alexithymia and relieving related psychological issues. We identified two systematic reviews on alexithymia by searching PubMed using the keywords ‘affective symptoms (MeSH)’ OR “Alexithymia^*^” AND “aged (MeSH)” OR “old people” OR “older people” OR “the elderly”. However, all the reviews focused on dialectical behavior therapy [15] and mindfulness-based treatment [16]. A systematic review of eight experimental studies involving 1148 participants ranging in age from 11 to 64 years, conducted by Salles et al. in 2023, showed that the overall intervention effect sizes on alexithymia could not be determined due to the lack of controlled trials, small sample sizes, and high variability of the DBT intervention. Another systematic review of four randomized control trials involving 460 participants with an average age of 23.5–53.8 years by Norman et al. in 2019 [15] showed a statistically significant effect of mindfulness-based treatment on alexithymia, whereas no significant effect was found at 2–3 months post-baseline. All of the studies included in the aforementioned systematic reviews focused on adolescents and adults, and the overall effects on alexithymia were not promising. In addition, the Toronto Alexithymia Scale, the common tool for assessing alexithymia, has been challenged by psychiatric comorbidities (e.g., depression, anxiety, sleep quality) due to the negative effects associated with self-criticism [17]. However, psychiatric comorbidities have been researched rarely to disprove the hypothesis that improvements in alexithymia are not caused by a reduction in negative effects. In light of the limitations of previous studies, it is necessary to conduct further research on effective interventions for alexithymia among older people in nursing homes.

The origins of alexithymia have long been controversial. There is some speculation that, individuals with alexithymia may concentrate attention on their physical symptoms and the medium’s effects due to the absence of a rich inner life, generating a paradigm of “operational thinking” that had a disastrous impact on their interpersonal skills [18]. Rational emotive behavior therapy (REBT), a type of cognitive behavioral therapy, is an action-oriented approach to assisting individuals in addressing their irrational beliefs or thoughts that cause their psychological confusion, and transforming them into healthy and rational cognition in order to treat their existing psychological problems [19]. According to REBT theory, changes to both rational and irrational beliefs result in changes to both dysfunctional emotions and maladaptive behaviors (outcomes). The goal of REBT is to convert irrational beliefs into rational beliefs. with a focus on evaluative beliefs. Individuals will constantly focus on and explore their inner world during this process, and “operational thinking” will be interrupted or stopped to the benefit of those who have alexithymia. A systematic review with meta-analysis of 80 studies from the last 50 years found that REBT was a long-lasting, safe therapy for various conditions, regardless of clinical status, sample age, or delivery format [20]. Therefore, we speculated that individuals with alexithymia who can concentrate on their mind may experience fewer psychosomatic symptoms, and that REBT can effectively improve alexithymia as well as related psychiatric comorbidities (i.e., depression, anxiety, sleep quality) of older people in nursing homes.

## Methods

The quasi-experimental study was conducted with a two-group pretest and posttest design. Due to a lack of cost and human resources [21], we employed convenience sampling and recruited approximately 30% of the older people from the two nursing homes involved in the study. However, other older people in the two nursing homes who were not recruited but may be eligible did not have the opportunity to participate in the random grouping process. To enhance the internal validity and credibility of the study, we randomly assigned 86 participants to two groups, the intervention group and the control group. The control group received usual care, while the intervention group received REBT intervention in addition to usual care. We followed the Transparent Reporting of Evaluations with Non-randomized Designs (TREND) reporting guidelines for this quasi-experimental study [22].

### Objectives

The objective was to evaluate the effects of REBT on alexithymia, anxiety, depression and sleep quality of older people in nursing homes. We assessed the effects within one-week post-intervention and at 3-month follow-up.

### Participants

We face to face recruited the older people living in two Changsha nursing homes in Hunan province, China, from March to May 2021. The two nursing homes selected by convenience sampling are both public facilities that provide both medical and nursing care. The number of older people in these two nursing homes is more than 200. One nursing home with 900 beds was the national demonstration nursing home, while another nursing home with 228 beds was rated 3 A in China, indicating they could provide standard care of a pretty high level. They have certain representations of nursing homes in China, regardless of their size and levels of care.

Inclusion criteria were: the elder aged ≥ 60 years old [23] with a Toronto Alexithymia Scale score of ≥ 61 indicating alexithymia [24]; good consciousness, no barriers in audio-visual, oral and other communication; do not leave the nursing homes during the research period, and be able to participate in weekly intervention. Exclusion criteria were: older people with major physical illnesses like cardiopulmonary failure or severe cognitive dysfunction like dementia; older people who have simultaneously participated in other research. Before the intervention, all study participants were invited to sign a written informed consent form after a team member explained the study’s goal and other pertinent information to them. All outcomes were assessed at baseline, within one-week post-intervention, and at the 3-month follow-up.

### Interventions

#### Intervention group procedure

The intervention group received REBT based on usual care in nursing homes. Three interventionists with standardized training who were under the supervision of a certified psychotherapist conducted structured REBT based on a manual approved by fifteen geriatric nurse specialists and psychologists. The interventionists were all registered nurses who have obtained a Bachelor’s degree in Nursing. An offline one-on-one interview was used for the intervention, which was conducted in the older people’ s solitary room. The older people’s intervention started within one week following the baseline data collection. The intervention took place for six weeks, once a week, for approximately 45 min each time. The first week of the intervention aimed to build a rapport of trust with older people and inform them know about the research plan. We would explain alexithymia to the older people. The second week’s intervention involved conducting a psychological assessment of the older people and determining the risks and manifestations of alexithymia in their daily lives. We would assess their knowledge of alexithymia. The third week’s intervention aimed to assist the older people recognize irrational beliefs. The fourth week of intervention aimed to guide the older people to debate with their irrational beliefs. The fifth week’s intervention aimed to encourage the older people to actively practice REBT in their daily lives. The sixth week’s intervention aimed to reinforce positive changes of the older people and deal with parting emotions. The REBT theme, objective and content for each week were shown in Additional file 1: Appendix A.

#### Control group procedure

The control group received usual care from uniformly trained nurses in nursing homes. Safe nursing requires maintaining the nursing home’s environmental safety, managing personnel effectively, preventing and controlling epidemics and guarding against older people suffering from acute illness, loss, fall, scald, electric shock and choking. Nurses should perform well in the surroundings of the nursing homes, provide older people with personal hygiene care, and pick up the trash once a day. Throughout the summer, they assist the older people in taking a daily bath and perform well in environmental sanitation and cleaning. In terms of life care, they should arrange for regular visits from family members and plan group activities for the older people, as well as supply the older people with services like diet, the purchase of vegetables and fruits, medication, personal cleanliness, life care, etc. The older people are scheduled for a physical examination during check-in in terms of medical care. Each room for the older people has call bells placed. There are medical rounds every day. The older people who are ill are promptly referred to hospitals for treatment, and following medical care, the older people are given care and follow-up.

### Outcomes

#### Social demographic questionnaire

The following questions are included in the questionnaire: gender, age, education level, length of stay in the nursing home, existence of the spouse, number of children, frequency of child visits, number of diseases, use of medications, participation in group activities in the nursing home, financial resources, and satisfaction with the nursing staff and the nursing home.

#### Toronto Alexithymia Scale (TAS-20)

The TAS-20 is a self-rating scale developed by Bagby et al. in 1994 [24]. It has three dimensions: emotional recognition disorder, emotional description disorder and externally-oriented thinking. There are 20 items total in this scale, of which item 1, 3, 6, 7, 9, 13, and 14 are the dimension of difficulty identifying feelings, item 2, 4, 11, 12, and 17 belong to the dimension of difficulty describing feelings, and item 5, 8, 10, 15, 16, 18, 19, and 20 are the dimension of externally-oriented thinking. The TAS-20 is currently the most widely used tool in the world to evaluate alexithymia. A total score of 61 points or above is regarded as alexithymia on this scale, which ranges from 20 to 100 points based on the Liket5 five-grade rating system. The Chinese TAS-20 scale has a Cronbach’s alpha coefficient of 0.83 and a retest reliability of 0.87 [25]. This scale is appropriate for the diagnosis of alexithymia in older people, according to prior research [10].

#### Patient Health Questionnaire Depression Module (PHQ-9)

The PHQ-9 scale, developed by Robort Spitzer in the 1990s, is widely used in general clinical diagnosis and depression screening [26]. The scale has 9 items, with a possible final score of 0 to 27. The severity of the depression increases with the scale’s overall score. The PHQ-9 scoring criteria states that a total score of < 5 indicates no depression while a score of ≥ 5 indicates depression. There are five different categories for the severity of depression: no depression (< 5), mild depression (5–9), moderate depression (10–14), moderately severe depression (15–19), and severe depression (≥ 20). Previous studies found that the PHQ-9, which has a satisfactory Cronbach’s alpha coefficient and split-half reliability (0.786, 0.676) of the Chinese version, is suitable for detecting depression in the Chinese older people [27].

#### Generalized anxiety Disorder-7 (GAD-7)

The GAD-7 was developed by Spitzer et al. in 2006 [28]. It has seven items with a total score of 0–21 points, with each item scoring 0–3 points. The severity of the anxiety increases with the scale’s overall score. The scoring standard divides the specific anxiety degree into four categories: no anxiety (< 4), mild anxiety (5–10), moderate anxiety (11–14), and severe anxiety (15–20). Previous research has demonstrated that the Chinese GAD-7’s Cronbach’s alpha coefficient is 0.90 and its retest reliability is 0.76 [29]. This scale is common and suitable for assessing anxiety in older people.

#### Pittsburgh Sleep Quality Index (PSQI)

The PSQI scale is widely used worldwide to evaluate sleep quality in the last month. It was developed by Buysse et al. in the United States in 1989 [30], and its reliability and validity were tested in China by Xian-chen Liu et al. (Liu et al., 1996) [31]. The Chinese version’s Cronbach’s α coefficient is 0.842. The PSQI consists of 19 items and 7 dimensions: subjective sleep quality, sleep latency, sleep duration, habitual sleep efficiency, sleep disturbance, sleep medication use, and daytime dysfunction. The total score ranges from 0 to 21, and as the score increases, the sleep quality decreases.

### Sample size

The effect size of REBT is approximately 0.70 [20], with *α* = 0.05 (two-sided) and *β* = 0.20. The sample size is *n1* = *n2* = 34 calculated by G-Power 3.1 software. Given the 20% lost-to-follow rate, this study includes 86 samples, with 43 in the intervention group and 43 in the control group.

### Assignment methods

The random grouping method of this study is as follows: First, SPSS 25.0 generates random numbers with a set beginning value of 200,000. The random numbers are distributed between 1 and 100, and are divided into groups of 1 and 2 using visual boxes. The intervention group was group 1, while the control group was group 2. The chosen list of the older people of nursing homes is ranked from 1 to 86 according to the initial order of surnames, and the ranking is contrasted with the random numbers generated using SPSS 25.0. If the number is 1, it will be added to the intervention group, and if it is 2, it will be added to the control group, in order to accomplish randomization.

### Blinding

The allocation was hidden from the nurses who gave older people their usual care. An assigned assessor who was not involved in the interventions and was blind to the participant randomization gathered all the data. A team member who was not directly involved in the data collecting carried out the data analysis.

### Unit of analysis

Since groups of individuals were assigned to the various research conditions, the analyses were carried out at the group level. To take into account the within-subject correlation, repeated measure ANOVA and generalized estimation equation were utilized.

### Statistical methods

The data were analyzed by SPSS 25.0 software. Descriptive analysis is used for the data. Counting data are represented by frequency and percentage, whereas normal data are represented by mean standard deviation and non-normal data are represented by median and interquartile spacing. Kolmogorov-Smirnov test was used for normality test. The independent sample T test, Mann-Whitney U test or chi-square test are used in the equilibrium test of baseline data for normally distributed data, non-normally distributed data, and counting data, respectively. The repeated measurement ANOVA can be used to test the changes of outcomes with normal distribution before and after intervention, while generalized estimating equation models can be used to assess the differential change in the outcomes between the two groups at T1 (within one-week post-intervention) and T2 (3-month follow-up) compared with T0 (baseline) for both outcomes with non-normally distributed outcomes. Simple effect analysis was used to decompose significant interactions into post hoc analysis. The test is bilateral, and a statistically significant difference is considered to exist at *P* < 0.05. During the research, try to keep the contact information of older people to prevent losing the visit. Two participants in this study were lost to follow-up (one moved out of the nursing home, and the other was referred for serious illness) with a low missing rate (2.33%), which can be considered as completely missing at random according to prior research [32]. It means that, notwithstanding the deletion of the missing data, the remaining complete sample can still accurately represent the entire sample.

## Results

### Participant flow and recruitment

**Fig. 1:**
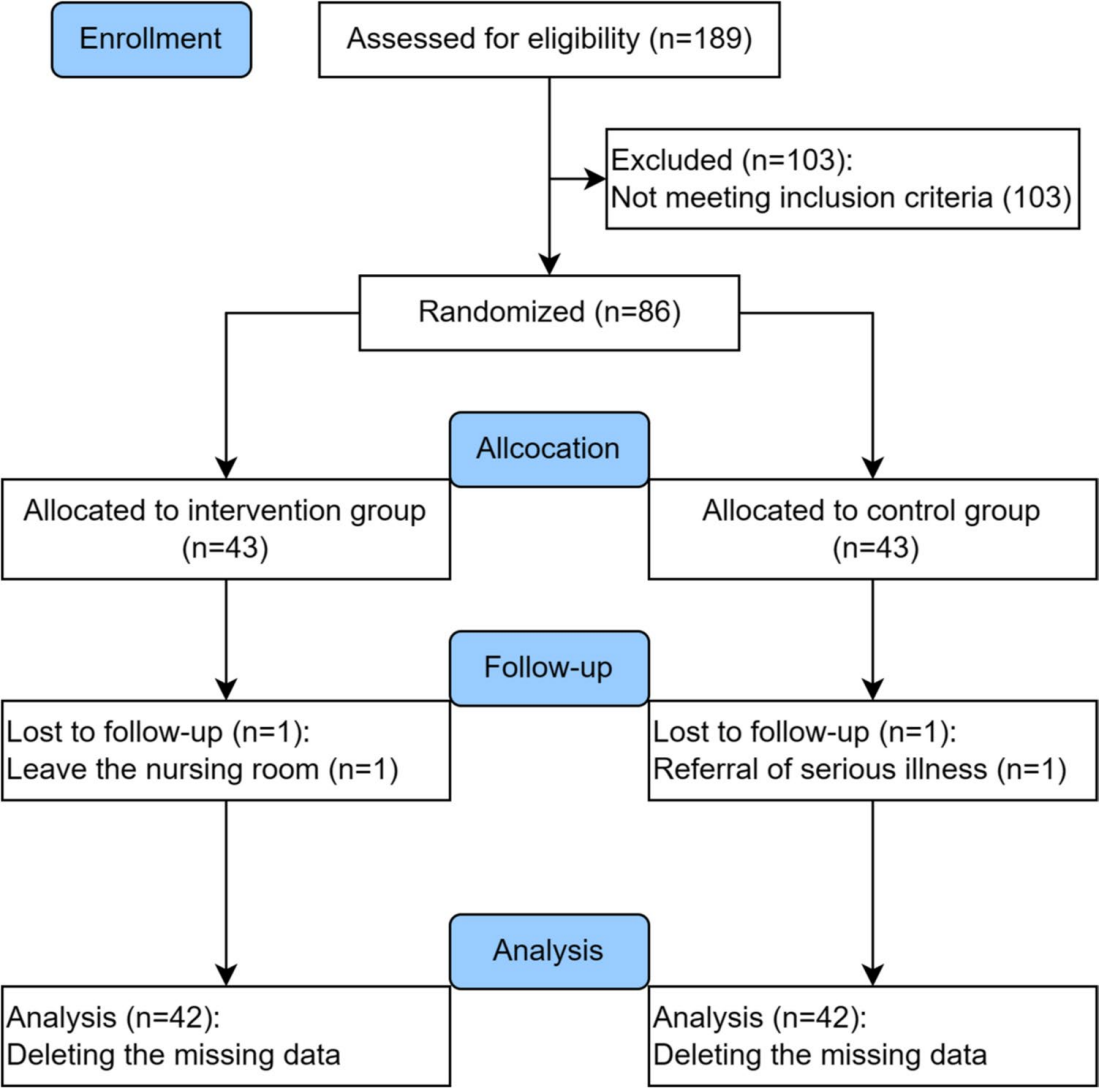
Flow diagram

A total of 338 older people were invited from March to May of 2021, and 189 of them were initially recruited at two nursing homes in Changsha with a high refuse rate of 44.1%. A total of 103 older people were excluded for not meeting the inclusion criteria. This study enrolled a total of 86 older people, with 43 assigned to the intervention group and 43 to the control group. After collecting baseline data in June 2021, one older people in the intervention group abruptly left the nursing home for family reasons throughout the intervention process. At the 3-month follow-up in November 2021, one older people in the control group was lost due to hospitalization for a serious illness. he overall lost visit rate was 2.3%, with two participants lost to follow-up. Regarding the missing data as completely missing at random, we deleted them from the final analysis of the data. More details were showed in Fig. 1.

### Baseline data and equivalence

This study included 84 older people in nursing homes whose ages ranged from 60 to 95 years, with 70.2% of them being female. More than three-quarters (67/79.8%) have a senior high school degree and above, and the majority (68/81.0%) have lived in nursing homes for more than six months. Only a few spouses of the older people (24/28.6%) are still alive. The average number of children among the older people is two. Nearly two-thirds of the older people’s children (55/65.5%) visit their parents in nursing homes at least twice a month. There are 62 older people (73.8%) suffering from two or more diseases. The majority of older people (75/89.3%) take medication daily. More than half (46/54.8%) are unwilling to participate in collective activities, and more than four-fifths (69/82.1%) have retirement wages. Almost of the older people (73/86.9%) are satisfied with nursing staff, and less than one-fifth (15/17.9%) are dissatisfied with the nursing homes in which they live.

Table 1 shows the demographic data of the older people in the intervention and control groups. The results of the equilibrium test show that the demographic data of the older people in the two groups are comparable (*P* > 0.05). The Kolmogorov-Smirnov test results for all baseline outcomes show significant differences (*P* < 0.05), indicating that all baseline outcomes were distributed non-normally. The results of generalized estimating equation (Table 2) show that no significant heterogeneity of the baseline outcomes between the intervention and control groups.

**Table 1:** Demographic data of the older people in intervention and control groups (*n* = 84)

Variables	Intervention group n(%)/M(Q1-Q3)	Control group n(%)/M(Q1-Q3)	*Z/χ* ^*2*^	*P*
**Gender**			1.424	0.233
Male	10(23.8)	15(35.7)		
Female	32(76.2)	27(64.3)		
**Age (year)**	85.0(80.0–88.0)	85.0(78.8–87.0)	-0.372^b^	0.710
**Degree of education**			0.664	0.415
Junior high school and below	10(23.8)	7(16.7)		
High school and above	32(76.2)	35(83.3)		
**Length of living in nursing home**			0.309	0.578
Within 6 months	9(21.4)	7(16.7)		
More than 6 months	33(78.6)	35(83.3)		
**Have a spouse or not**			0.233	0.629
Yes	13(31.0)	11(26.2)		
No	29(69.0)	31(73.8)		
**Number of children**	2(1.8-3.0)	2(2.0–3.0)	-1.392^b^	0.164
**Frequency of family visits**			1.252	0.741
< 1 /month	8(19.0)	7(16.7)		
1 /month	6(14.3)	8(19.0)		
2–4 /month	19(45.2)	15(35.7)		
5 and above/month	9(21.4)	12(28.6)		
**Number of illnesses**			0.000	1.000
1 and below	11(26.2)	11(26.2)		
2 and above	31(73.8)	31(73.8)		
**Take medicine or not**			0.498^a^	0.480
Yes	39(92.9)	36(85.7)		
No	3(7.1)	6(14.3)		
**Participate in group activities**			0.769	0.381
Yes	21(50.0)	17(40.5)		
No	21(50.0)	25(59.5)		
**Source of income**			0.081	0.776
Retirement wages	34(81.0)	35(83.3)		
Others	8(19.0)	7(16.7)		
**Satisfaction with nursing staff**			0.105	0.746
Yes	37(88.1)	36(85.7)		
No	5(11.9)	6(14.3)		
**Satisfaction with nursing home**			0.081	0.776
Yes	34(81.0)	35(83.3)		
No	8(19.0)	7(16.7)		

^a^Continuity correction for chi-square test

^b^ the Mann-Whitney U test; M(Q1-Q3), medians (quartile 1 to quartile 3)

**Table 2:** Effects of rational emotive behavior therapy on alexithymia, depression, anxiety and sleep quality of the elderly in nursing homes (*n* = 84)^a^

Outcome	M(Q1-Q3)		Group Effect ^**b**^		Time Effect ^**c**^		Group × Time Effect ^**d**^	
	Intervention group	Control group	β (95%***CI***)	***P***	β(95%***CI***)	***P***	β (95%***CI***)	***P***
**TAS-20** ^**e**^								
T0	68.00 (62.00-77.25)	69.00 (62.00-75.00)	0.929 (-2.402 to 4.260)	0.585	NA	NA	NA	NA
T1	59.00 (55.75-63.00)	66.50 (60.75-74.25)			-2.000 (-2.457 to -1.543)	<0.001	-8.167 (-10.965 to -5.368)	<0.001
T2	64.50 (61.00-69.5)	69.50 (63.00-76.00)			0.429 (0.005 to 0.852)	0.047	-4.119 (-7.171 to -1.067)	0.008
**PHQ-9** ^**f**^								
T0	6.00 (3.75-12.25)	6.00 (3.75-11.25)	0.262 (-1.490 to 2.014)	0.770	NA	NA	NA	NA
T1	6.00 (3.75-9.25)	6.00 (2.00-11.00)			-1.119 (-2.446 to 0.208)	0.098	-0.429 (-3.081 to 2.224)	0.751
T2	6.50 (2.00-10.25)	6.00 (2.00-11.00)			-0.905 (-2.799 to 0.989)	0.349	-0.429 (-4.216 to 3.358)	0.824
**GAD-7** ^**e**^								
T0	4.50 (1.00-9.00)	3.00 (1.00-7.25)	1.048 (-1.076 to 3.172)	0.334	NA	NA	NA	NA
T1	4.00 (1.00-7.00)	3.00 (2.00-7.25)			0.357 (0.103 to 0.611)	0.006	-0.500 (-2.288 to 1.288)	0.584
T2	3.00(1.00-8.00)	4.50 (1.00-8.00)			0.357 (-1.705 to 2.420)	0.734	-0.167 (-2.119 to 1.786)	0.867
**PSQI** ^**e**^								
T0	9.00 (5.00-15.00)	8.50 (4.75-13.00)	0.405 (-1.679 to 2.489)	0.703	NA	NA	NA	NA
T1	7.00 (5.00-11.00)	9.00 (5.75-14.00)			0.881 (0.592 to 1.170)	<0.001	-1.619 (-3.533 to 0.295)	0.097
T2	7.00 (5.00-11.00)	10.00 (5.75-14.50)			1.333 (0.962 to 1.705)	<0.001	-2.048 (-4.004 to -0.091)	0.040

*Abbreviations: M(Q1-Q3)* medians (quartile 1 to quartile 3), *CI* Confidence Interval, *TAS-20* Toronto Alexithymia Scale, *PHQ-9* Patient Health Questionnaire Depression Module, *GAD-7* Generalized Anxiety Disorder-7, *PSQI* Pittsburgh Sleep Quality Index, *T0* baseline, *T2* within one-week post-intervention, *T3* 3-month follow-up, *NA* not applicable

^a^The control group and T0 were the reference categories in the generalized estimating equation model and its corresponding null variables

^b^Group effect was defined as group differences between the intervention and control groups at T0

^c^Time effect was defined as the change in scores at T1, T2 compare to T0 separately. When interactions are significant, it refers to the change in scores for the control group

^d^Group × time effect was defined as additional change in scores for the intervention group compared to the control group at T1 and T2 separately

^e^Significant interactions were followed with simple effects analyses

^f^The main effects were assessed when interactions were not significant

### Numbers analyzed

The analysis included 84 participants who completed the intervention and 3-month follow-up. There is no intention-to‐treat analysis in the study due to a low amount of completely random missing data [33].

### Outcomes and estimation

For alexithymia, the two groups differed significantly at both T1 and T2. The intervention group shows a significantly greater improvement in alexithymia than the control group (T1: *β* = -8.167, 95%*CI*= -10.965, -5.368, *P* < 0.001; T2: *β*= -4.119, 95%*CI*= -7.171, -1.067, *P* = 0.008). In the control group, alexithymia improved significantly at both T1 and T2 (T1: *β* = -2.000, 95%*CI*= -2.457, -1.543, *P* < 0.001; T2: *β* = 0.429, 95%*CI*= -0.005, 0.852, *P* = 0.047). For alexithymia dimensions, the two groups showed significant differences at both T1 and T2 in both difficulty identifying feelings (T1: *β* = -5.405, 95%*CI*= -7.125, -3.684, *P* < 0.001; T2: *β*= -2.357, 95%*CI*= -4.252, -0.463, *P* = 0.015) and difficulty describing feelings (T1: *β* = -2.071, 95%*CI*= -3.219 to -0.924, *P* < 0.001; T2: *β*= -1.333, 95%*CI*= -2.653 to -0.014, *P* = 0.048).

For depression and anxiety, no significant differences were noted between the intervention and control groups at T1 and T2. There were no significant improvements were found in depression for either group at T1 and T2. The anxiety in the intervention group has a significantly greater improvement at T1 compared to T0 (T1: *β* = -1.190, 95%*CI*= -1.961, -0.420, *P* = 0.002), but in the control group it worsens significantly (T1: *β* = 0.357, 95%*CI* = 0.103, 0.611, *P* = 0.006).

Compared to the control group, the intervention group shows a significant improvement in sleep quality at T2 (*β* = -2.048, 95%*CI*=-4.004, -0.091, *P* = 0.040). Sleep quality worsens significantly in the control group at both T1and T2 compared to T0 (T1: *β* = 0.881, 95%*CI* = 0.592, 1.170, *P* < 0.001; T2: *β* = 1.333, 95%*CI* = 0.962, 1.705, *P* < 0.001), but it has a significantly greater improvement in the intervention group (T1: *β*= -1.143, 95%*CI*=-1.861, -0.424, *P* = 0.002; T2: *β*= -1.119, 95%*CI*= -1.793, -0.445, *P* = 0.001). For sleep quality dimensions, the two groups showed significant differences at both T1 and T2 in both sleep disturbance (T1: *β*= -0.333, 95%*CI*= -0.504, -0.163, *P* < 0.001; T2: *β*= -0.381, 95%*CI*= -0.567, -0.195, *P* < 0.001) and daytime dysfunction (T1: *β*= -0.524, 95%*CI*= -0.901, -0.146, *P* = 0.007; T2: *β*= -0.619, 95%*CI*= -0.980 to -0.258, *P* = 0.001). See more details in Table 2. Additional file 2: Appendix B shows the effects of REBT on alexithymia and sleep quality dimensions. Spearman correlation analysis revealed significant correlations between the decrease in scores of alexithymia dimensions and the improvement in sleep quality scores (Table 3).

**Table 3:** Correlation between decreased scores of alexithymia dimensions and improvement in sleep quality scores before and after rational emotive behavior therapy among older people (*n* = 42)

	1	2	3	4
1 Difficulty identifying feelings	-			
2 Difficulty describing feelings	0.512^***^	-		
3 Externally-oriented thinking	0.025	0.361^**^	-	
4 Alexithymia	0.649^***^	0.832^***^	0.560^***^	-
5 Sleep quality	0.455^***^	0.316^**^	0.277^*^	0.441^***^

^***^ *P* < 0.001; ^**^ *P* < 0.01; ^*^ *P* < 0.05

### Ancillary analyses and adverse events

No ancillary analyses were undertaken. There were no adverse events reported.

## Discussion

To our knowledge, this is the first study to evaluate the effects of REBT on alexithymia, anxiety, depression and sleep quality of older people in nursing homes. The results showed that REBT can not only effectively improve alexithymia of older people in nursing homes but also has a positive effect on sleep quality. However, there were no statistically significant differences in anxiety and depression between the two groups.

The study found that the intervention group shows a significantly greater improvement in alexithymia than the control group after the intervention and at the 3-month follow-up. Our study was the first to conduct REBT with a focus on alexithymia of older people in nursing homes, and it added new knowledge about how to address older people’s alexithymia. Norman et al [16] showed that mindfulness-based treatment has a statistically significant effect on alexithymia in the non-older people compared to the control group, but no statistically significant differences at 2-3 months post-baseline. REBT appears to be more successful than mindfulness-based treatment in the short-term alleviation of alexithymia. Previous study has shown that people with alexithymia and healthy people have different beliefs regarding emotionally induced events, with those with alexithymia being more prone to hold negative beliefs about past events and interpret the present fatalistically [34]. Although there is no definitive conclusion about the mechanism of alexithymia, a number of studies have demonstrated that people with alexithymia have a degree of cognitive impairment [35, 36], particularly a lack of cognitive flexibility, which makes it difficult to appropriately identify and give feedback to social emotions [37]. From the perspective of REBT mechanism of action, REBT can change the irrational beliefs of older people by intervening with their cognition and belief, so reducing their negative feelings about life events, thereby enabling a fundamental change in the occurrence and development of alexithymia. However, alexithymia rebounded in both groups at 3-month follow-up, suggesting that the long-term effect of REBT on alexithymia may need further evaluation. Furthermore, REBT can effectively improve difficulty in identifying and describing feelings. This could be attributed to the fact that REBT can help older people address irrational beliefs and cognitive distortions. They were encouraged to explore their emotions, overcome barriers to recognizing feelings, and learn better ways to express themselves, leading to an enhanced ability to identify and describe their feelings effectively [19]. However, externally oriented thinking cannot be significantly improved after REBT. This could be because older people with high levels of externally oriented thinking tend to rely less on confrontive coping strategies [38], while REBT can target cognitive distortions and emotional regulation, it may not directly address coping styles related to external orientation. Thus, combining REBT with complementary interventions that target coping changes may lead to more comprehensive improvements in externally oriented thinking.

This study showed that the sleep quality among older people in nursing homes was poor, which is consistent with recent studies indicating that the prevalence of poor sleep quality was relatively high among the same population in China [39]. The double changes in physiology and environment may explain why older people in nursing homes have poor sleep quality. On the one hand, older people in nursing homes cannot live with their families and are more likely to have inadequate social support than the general older people population [40], which may lead to poor sleep quality [41]. On the other hand, as older people age, their bedtime and wake-up time will be advanced, while they are instructed to adhere to the nursing homes’ regular management, which may alter the older people’s sleep habits and result in sleep fragmentation. Fortunately, REBT appeared to be an effective method for improving the sleep quality of older people in nursing homes, particularly in addressing sleep disturbance and daytime dysfunction. The finding showed the intervention group had better sleep quality than the control group at 3-month follow-up, and significant within-group improvements for the intervention group and within-group deterioration for the control group were observed within one-week post-intervention and at 3-month follow‐up. The findings were somewhat similar to those of a previous study by Kim et al [42], who found similar changes in renal dialysis patients, i.e., significant differences in sleep quality between the groups were observed until the 3-month follow‐up. Such changes could be caused by direct and indirect factors. Directly, REBT has shown the potential to partially modify older people's misconceptions about sleep problems, reduce pre-bedtime stress, and promote relaxation before sleep, resulting in improvements in sleep disturbance and daytime dysfunction [43]. Consequently, the overall sleep quality of older people in nursing homes was significantly enhanced. Indirectly, this result may be explained by the time lags of effects of REBT. A systematic review revealed a strong association between alexithymia and sleep problems [12]. With the improvement of alexithymia, older people may experience reduced susceptibility to negative emotions and enhanced emotional regulations, attributed to the reduction of irrational beliefs [19]. The positive change can contribute to a more relaxed state of mind before bedtime, gradually leading to the improvements in sleep disturbance and daytime dysfunction [44, 45]. As a result, the overall sleep quality was enhanced, benefiting older people in nursing homes.

Previous longitudinal study has found the associations between alexithymia and anxiety [11]. A systematic review including 3572 participants also revealed that alexithymia assessed by TAS-20 is closely related to depression [46]. These correlations meant that improvements in alexithymia may alleviate anxiety and depression. REBT has been found to have a significant effect on the control of depression and anxiety in adolescents [47], college students [48] and adults with congenital heart disease [49]. However, the results showed that REBT failed to significantly alleviate anxiety and depression of older people in nursing homes. Two major reasons may explain the finding. First, according to the scoring criteria of the PHQ-9 and GAD-7 [26, 28], low anxiety and depression levels at baseline in both groups may impede the detection of a significant effect of REBT. Second, the intervention length and frequency were similar to previous studies in general population [47–49]. Due to decreased cognition and inability to acquire new skills in older people [50], the same intervention length and frequency may not have the similar effect on anxiety and depression in older people as it does in younger individuals. Nevertheless, no statistically significant differences in the improvement of anxiety and depression can support the hypothesis that improvements in alexithymia are not induced by a reduction in anxiety and depression.

### Limitations

Firstly, we did not screen all older people in the two nursing homes due to the limitation of cost and human resources. The quasi-experimental design of this study is less robust than randomized controlled trials. Secondly, the follow-up visit lasted only three months and did not assess the long-term effects. Then, the study was conducted in Changsha, China. How sustainable and generalizable these interventions are beyond the research setting needs to be determined. Last but most importantly, the scores of alexithymia, depression, and anxiety were slightly above the cut-off scores, indicating the possibility of a floor effect that cannot be overlooked. This floor effect might have contributed to the non-significant effects of REBT on improving depression and anxiety in this study. For future research, it is recommended to recruit a larger sample with higher scores of alexithymia, depression, and anxiety, and utilize more sensitive measurement tools to gain a more accurate understanding of the REBT's effects. This approach can help overcome the limitations of the floor effect and provide more robust findings regarding the effectiveness of REBT in addressing depression and anxiety among older people.

## Conclusion

The findings revealed that REBT was an effective way to improve alexithymia and sleep quality of older people in nursing homes. We recommended it as a useful and straightforward method for nursing home nurses, providing them with training in simplified REBT techniques or strategies. These techniques can be integrated into their interactions with older people to offer enhanced mental health care and support. However, REBT fails to significantly improve depression and anxiety of older people in nursing homes. Future research is need to emphasize further improvement mechanisms of REBT and optimize this intervention to enhance the short-term effects and maintain long-term effects.

### Supplementary Information

**Additional file 1: Appendix A.** Rational emotive behavior therapy theme, objective and content of each week

**Additional file 2: Appendix B.** Effects of rational emotive behavior therapy on alexithymia and sleep quality dimensions of older people in nursing homes (*n*=84)

## Data Availability

The datasets used and/or analyzed during the current study are available from the corresponding author on reasonable request.
