# Supplementary material for: Effects of rational emotive behavior therapy on alexithymia, anxiety, depression and sleep quality of older people in nursing homes: a quasi-experimental study
Source: BMC Nurs. 2023 Aug 24;22:280. doi: 10.1186/s12912-023-01449-9 (PMC10463404; doi:10.1186/s12912-023-01449-9)
Supplement: Supplementary file 1 — Additional file 1: Appendix A. Rational emotive behavior therapy theme, objective and content of each week [file 12912_2023_1449_MOESM1_ESM.pdf]

Appendix A Rational emotive behavior therapy theme, objective and content of each week

Theme	Objective	Content
First week: establish a trust relationship	1. Introduction.<br>2. Introduce the purpose and content of the intervention and deliver its booklet.<br>3. Explain the definition, assessment and adverse effects of alexithymia.<br>4. Make an appointment for the next visit.	1. Introduction.<br>2. Expresses understanding of the study content and alexithymia.<br>3. Express willingness and expectation to cooperate with the intervention.<br>4. Determine relevant details for the next visit.
Second week: conduct psychological diagnosis	1. Ensure that the elderly understand alexithymia.<br>2. Identify existing problems and their potential causes, classify these problems, and make notes to determine the most pressing problem to solve with the elderly.<br>3. Make an appointment for the next visit.	1. Explain alexithymia on their own.<br>2. Describe life events related to alexithymia, depression, anxiety, sleep quality, etc., and the psychological process that occurred during the event.<br>3. Express the desire to resolve certain psychological problems.<br>4. Determine relevant details for the next visit.
Third week: distinguish irrational beliefs	1. Review the events and psychological problems mentioned the prior week.<br>2. Explain the irrational beliefs and give examples.<br>3. Identify irrational beliefs expressed by the elderly.<br>4. Make an appointment for the next visit.	1. Discuss the events and psychological problems mentioned the prior week.<br>2. Understand the irrational beliefs.<br>3. Explore their own irrational beliefs in light of experiences.<br>4. Express the willingness to change irrational beliefs.<br>5. Determine relevant details for the next visit.
Fourth week: debate with irrational beliefs	1. Review relevant events and irrational beliefs, and guide the elderly in dissecting and refuting them. Other cases can be used to identify and refute the claim.<br>2. Evaluate their ability to identify and	1. Discuss last week's described events and irrational beliefs.<br>2. Acquire the skill of debating irrational beliefs and gain experience via practice (my own example, other cases, videos, etc.).

	debate irrational beliefs, and encourage them to apply the method to improve their mental health and more openly express their feelings and needs in everyday life.<br>3. Set personalized homework with the elderly and encourage them to complete it.<br>4. Make an appointment for the next visit.	3. Express a willingness to apply the debate method in everyday life.<br>4. Understand and finish the assigned homework.<br>5. Determine relevant details for the next visit.<br>Assignment 1: Apply the method in your daily life and share your experience the next week.
Fifth week: make positive changes	1. Examine the assigned homework, determine the difficulties in completing it, and assess its completion.<br>2. Provide reinforcement for the difficulties, encourage the elderly to persist in applying and changing their way of thinking and behaving, and apply the method to various emotional events.<br>3. Assign new homework.<br>4. Make an appointment for the next visit.	1. Show the homework and emphasize its difficulty.<br>2. Explore the process of debating irrational beliefs in depth.<br>3. Express the willingness to actively apply the method in everyday life.<br>4. Understand and complete the new homework.<br>5. Determine relevant details for the next visit.<br>Assignment 2: Develop a behavioral plan for coping with irrational beliefs and consider what to show with interventionists.
Sixth week: reinforce positive changes and keep a pleasant parting	1. Examine the assigned homework and assess its completion.<br>2. Ask the elderly about their difficulties and concerns and assist them in resolving them; assess the ability to identify and refute irrational beliefs through real-world cases.<br>3. Explain some everyday healthcare techniques for physical illnesses.<br>4. Encourage the elderly to use REBT in their daily lives.	1. Demonstrate and discuss the homework with the interventionists.<br>2. Describe and address any application-related concerns.<br>3. Acquire some healthcare techniques for physical illnesses.<br>4. Express a willingness to apply the method in everyday life.<br>5. Express the emotion of separation and deal with it positively.

	5. Express well wishes and address parting feelings.	
