# Supplementary material for: Effects of rational emotive behavior therapy on alexithymia, anxiety, depression and sleep quality of older people in nursing homes: a quasi-experimental study
Source: BMC Nurs. 2023 Aug 24;22:280. doi: 10.1186/s12912-023-01449-9 (PMC10463404; doi:10.1186/s12912-023-01449-9)
Supplement: Supplementary file 2 — Additional file 2: Appendix B. Effects of rational emotive behavior therapy on alexithymia and sleep quality dimensions of older people in nursing homes (n=84) [file 12912_2023_1449_MOESM2_ESM.pdf]

Appendix B Effects of rational emotive behavior therapy on alexithymia and sleep quality dimensions of older people in nursing homes (n=84)

Outcome	M(Q1-Q3)		Group Effect [b]		Time Effect [c]		Group × Time Effect [d]	
	Intervention group	Control group	β (95%*CI*)	*P*	β(95%*CI*)	*P*	β (95%*CI*)	*P*
**TAS-20_ difficulty identifying feelings [e]**								
T0	23.00 (21.00-26.25)	22.50(20.00-26.25)			NA	NA	NA	NA
T1	17.00(15.00-20.00)	22.00(20.00-26.25)	0.595(-1.332 to 2.522)	0.545	-0.571(-0.860 to -0.283)	<0.001	-5.405(-7.125 to -3.684)	<0.001
T2	20.50(18.00-23.00)	23.00(20.00-26.25)			0.262(0.075 to 0.449)	0.006	-2.357(-4.252 to -0.463)	0.015
**TAS-20_ difficulty describing feelings [e]**								

T0	17.00 (15.75-21.00)	17.00(15.00-20.25)			NA	NA	NA	NA
T1	14.50(13.00-16.25)	16.50(15.00-20.00)	0.500(-0.784 to 1.784)	0.446	-0.690(-1.030 to -0.351)	<0.001	-2.071(-3.219 to -0.924)	<0.001
T2	16.00(14.75-18.25)	16.50(15.00-21.00)			0.071(0.067 to 0.210)	0.311	-1.333(-2.653 to -0.014)	0.048
**TAS-20_ externally-oriented thinking [f]**								
T0	28.00 (25.00-32.00)	28.00(26.00-31.00)			NA	NA	NA	NA
T1	27.00(25.00-28.25)	28.00(25.00-30.00)	-0.429(-1.562 to 0.705)	0.459	-1.000(-1.837 to -0.163)	0.019	-0.524(-2.194 to 1.146)	0.539
T2	28.00(26.00-29.00)	28.50(26.00-31.00)			-0.036(-0.826 to 0.754)	0.929	-0.262(-1.841 to 1.317)	0.745

**PSQI- subjective sleep quality [e]**								
T0	2.00 (1.00-2.00)	1.00(1.00-2.00)			NA	NA	NA	NA
T1	1.00(1.00-2.00)	1.00(1.00-2.00)	0.190(-0.127 to 0.508)	0.240	0.000(0.000 to 0.000) [g]	1.000	-0.071(-0.382 to 0.239)	0.652
T2	1.00(1.00-2.00)	1.00(1.00-2.00)			0.048(-0.017 to 0.112)	0.147	-0.167(-0.483 to 0.149)	0.301
**PSQI- sleep latency [e]**								
T0	1.50(1.00-2.00)	2.00(0.00-2.00)			NA	NA	NA	NA
T1	1.00(1.00-2.00)	1.00(0.00-2.00)	0.119(-0.325 to 0.563)	0.599	0.000(0.000 to 0.000) [g]	<0.001	-0.119(-0.531 to 0.293)	0.571

T2	1.00(1.00-2.00)	1.00(0.00-2.25)			0.071(-0.031 to 0.174)	0.170	-0.190(-0.614 to 0.233)	0.379
**PSQI- sleep duration [e]**								
T0	1.00(0.00-2.00)	1.00(0.00-2.00)			NA	NA	NA	NA
T1	1.00(0.00-2.00)	2.00(0.00-3.00)	0.095(-0.409 to 0.599)	0.711	0.357(0.185 to 0.530)	<0.001	-0.405 (-0.923 to 0.113)	0.126
T2	1.00(0.00-2.00)	2.00(0.00-3.00)			0.405 (0.229 to 0.580)	<0.001	-0.452(-0.971 to 0.066)	0.087
**PSQI- habitual sleep efficiency [e]**								
T0	1.00(0.00-2.25)	1.00(0.00-2.00)	0.095(-0.411 to 0.601)	0.712	NA	NA	NA	NA

T1	1.00(0.00-2.00)	1.00(0.00-2.00)			0.071(-0.031 to 0.174)	0.170	-0.071(-0.567 to 0.424)	0.778
T2	1.00(0.00-2.00)	1.00(0.00-2.00)			0.071(-0.031 to 0.174)	0.170	-0.119(-0.616 to 0.378)	0.638
**PSQI- sleep disturbance** [e]								
T0	1.00(1.00-1.00)	1.00(1.00-1.00)			NA	NA	NA	NA
T1	1.00(1.00-1.00)	1.00(1.00-2.00)	-0.095(-0.222 to 0.032)	0.141	0.214(0.074 to 0.355)	0.003	-0.333(-0.504 to -0.163)	<0.001
T2	1.00(1.00-1.00)	1.00(1.00-2.00)			0.262(0.113 to 0.410)	0.001	-0.381(-0.567 to -0.195)	<0.001
**PSQI- sleep medication use** [e]								
T0	0.00(0.00-2.00)	0.00(0.00-2.25)	0.048(-0.500 to 0.595)	0.865	NA	NA	NA	NA

T1	0.00(0.00-2.00)	0.00(0.00-3.00)			0.857(0.447 to 1.267)	<0.001	-0.095(-0.633 to 0.442)	0.728
T2	0.00(0.00-2.00)	0.00(0.00-3.00)			0.976(0.556 to 1.396)	<0.001	-0.119(-0.687 to 0.449)	0.681
**PSQI- daytime dysfunction [e]**								
T0	2.00(1.00-3.00)	2.00(1.00-3.00)			NA	NA	NA	NA
T1	2.00(1.00-2.00)	2.00(1.75-3.00)	-0.048(-0.437 to 0.342)	0.811	2.167(1.895 to 2.438)	<0.001	-0.524(-0.901 to -0.146)	0.007
T3	2.00(1.00-2.00)	2.50(2.00-3.00)			2.286(2.045 to 2.526)	<0.001	-0.619(-0.980 to -0.258)	0.001

Abbreviations: M(Q1-Q3), medians (quartile 1 to quartile 3); CI, Confidence Interval; TAS-20, Toronto Alexithymia Scale; PHQ-9, Patient Health Questionnaire Depression Module; GAD-7, Generalized Anxiety Disorder-7; PSQI, Pittsburgh Sleep Quality Index; T0, baseline; T2, within one-week post-intervention; T3, 3-month follow-up; NA, not applicable.

a. The control group and T0 were the reference categories in the generalized estimating equation model and its corresponding null variables.

b. Group effect was defined as group differences between the intervention and control groups at T0.

c. Time effect was defined as the change in scores at T1, T2 compare to T0 separately. When interactions are significant, it refers to the change in scores for the control group.

d. Group × time effect was defined as additional change in scores for the intervention group compared to the control group at T1 and T2 separately.

e. Significant interactions were followed with simple effects analyses.

f. The main effects were assessed when interactions were not significant.

g. The result is scientific counting, which is very close to 0, expressed by 0.000.
